# Associations of Sarcopenia and Its Components With Cardiovascular Risk: Five‐Year Longitudinal Evidence From China Health and Retirement Longitudinal Study

**DOI:** 10.1161/JAHA.124.040099

**Published:** 2025-06-18

**Authors:** Yang Chen, Ziyi Zhong, Konstantinos Prokopidis, Ying Gue, Garry McDowell, Yang Liu, Coleen Ditchfield, Muath Alobaida, Bi Huang, Gregory Y. H. Lip

**Affiliations:** ^1^ Liverpool Centre for Cardiovascular Science University of Liverpool, Liverpool John Moores University and Liverpool Heart & Chest Hospital Liverpool United Kingdom; ^2^ Department of Cardiovascular and Metabolic Medicine, Institute of Life Course and Medical Sciences University of Liverpool United Kingdom; ^3^ Department of Musculoskeletal Ageing and Science, Institute of Life Course and Medical Sciences University of Liverpool United Kingdom; ^4^ School of Pharmacy and Biomolecular Sciences Liverpool John Moores University Liverpool United Kingdom; ^5^ Department of Cardiovascular Medicine The Second Affiliated Hospital, Jiangxi Medical College, Nanchang University Nanchang Jiangxi China; ^6^ Department of Medicine for Older People Whiston Hospital, Mersey and West Lancashire Teaching Hospitals NHS Trust Prescot United Kingdom; ^7^ Department of Basic Science Prince Sultan Bin Abdulaziz College for Emergency Medical Services, King Saud University Riyadh Saudi Arabia; ^8^ Department of Cardiology the First Affiliated Hospital of Chongqing Medical University Chongqing China; ^9^ Danish Center for Health Services Research, Department of Clinical Medicine Aalborg University Aalborg Denmark

**Keywords:** 5‐time chair stand test, all‐cause mortality, cardiovascular disease, heart disease, sarcopenia, stroke, Cardiovascular Disease, Risk Factors, Primary Prevention, Clinical Studies

## Abstract

**Background:**

Sarcopenia, an age‐related condition, has an unclear association with cardiovascular disease (CVD) risk. We aimed to analyze whether sarcopenia and its individual components are associated with new‐onset CVD in middle‐aged and older adults.

**Methods and Results:**

Data were derived from the China Health and Retirement Longitudinal Study, with sarcopenia defined by the Asian Working Group for Sarcopenia 2019 criteria. The primary outcome was composite CVD, comprising heart disease and stroke. Multivariable Cox proportional hazards regression analysis and Fine–Gray subdistribution hazards models were used to calculate hazard ratios (HRs), subdistribution hazard ratios (SHRs), and 95% CIs. A total of 10 649 participants (mean age 64.5±10.7 years, 47.6% male) were included. During mean follow‐up of 4.60±1.06 years, there were 1649 (15.5%) cases of new‐onset CVD. Possible sarcopenia was linked to increased new‐onset composite CVD risk (HR, 1.21 [95% CI, 1.06–1.37]; SHR, 1.20 [95% CI, 1.05–1.35]), whereas sarcopenia and severe sarcopenia showed no association. Restricted cubic spline analysis revealed that 5‐time chair stand test (5‐CST) was associated with new‐onset composite CVD, with significant sex‐specific interaction (*P*‐for‐interaction=0.001). Compared with 5‐CST≤9.0 s, higher risk of new‐onset composite CVD was observed in men for 9.0 s<5‐CST≤15.0 s (HR, 1.36 [95% CI, 1.16–1.59]; SHR, 1.34 [95% CI, 1.15–1.56]) and 5‐CST>15.0 s (HR, 2.19 [95% CI, 1.65–2.90]; SHR, 2.00 [95% CI, 1.53–2.63]). Among women, 5‐CST>8.5 s had higher risk of new‐onset composite CVD compared with 5‐CST≤8.5 s (HR, 1.26 [95% CI, 1.09–1.45]; SHR, 1.25 [95% CI, 1.09–1.43]).

**Conclusion:**

Possible sarcopenia was associated with increased risk of new‐onset composite CVD, suggesting that progression to definite sarcopenia may not parallel cardiovascular risk. Longer 5‐CST was linked to higher risk of new‐onset composite CVD, with sex‐specific association.

**Nonstandard Abbreviations and Acronyms**
5‐CST: 5‐time chair stand test
6‐WT: 6‐meter walking speed test
ASM: appendicular skeletal muscle mass
AWGS: Asian Working Group on Sarcopenia
CHARLS: China Health and Retirement Survey
HGS: handgrip strength
SMI: skeletal muscle index

**Clinical Perspective**

**What Is New?**

- In a large cohort of 10 649 middle‐aged and older adults from the China Health and Retirement Longitudinal Study, possible sarcopenia—but not sarcopenia or severe sarcopenia—was associated with an increased risk of new‐onset composite cardiovascular disease.
- Longer 5‐time chair stand test, indicating lower physical performance, associated with higher risk of new‐onset composite cardiovascular disease, with sex‐specific thresholds: 9.0 and 15.0 s for men, and 8.5 s for women.

**What Are the Clinical Implications?**

- Early declines in muscle function, rather than advanced sarcopenia, may be strongly related to higher risk of new‐onset composite cardiovascular disease, highlighting the need for early screening and intervention at the stage of possible sarcopenia.
- Five‐time chair stand test as a functional assessment could enhance cardiovascular risk stratification, with sex‐specific cutoffs guiding personalized prevention strategies for middle‐aged and older adults.

Sarcopenia is a syndrome associated with aging or secondary to other diseases, characterized by loss of muscle mass, strength, and function, associated with malnutrition or lack of physical activity.^1^ This condition significantly increases the risk of several adverse outcomes, including fractures and falling,^2^ frailty and disability,^3^ and mortality.^4^ Currently, various organizations, including the Foundation for the National Institutes of Health in the United States,^5^ the European Working Group on Sarcopenia in Older People,^6^ and the Asian Working Group on Sarcopenia (AWGS),^7^ have proposed diverse diagnostic criteria for sarcopenia, resulting in reported prevalence rates ranging from 10% to 27% across different regions.^8^

Globally, the older population has reached approximately 700 million in 2019, with an anticipated 2‐fold increase by 2050, and a 3‐fold rise in older adults aged >80.^9^ As age is a major risk factor for cardiovascular disease (CVD), this demographic shift is likely to drive a corresponding rise in CVD prevalence.^10^ In China, the population with CVD grew from 40.6 to 93.8 million between 1990 and 2016.^11^ Given these trends, it is imperative to investigate risk factors contributing to CVD occurrence, facilitating the development of targeted and effective preventive and intervention strategies aimed at mitigating the health care burden of CVD.

Emerging evidence suggests a strong association between sarcopenia and CVD, as both conditions share common health complications related to aging, such as skeletal muscle insulin resistance and chronic inflammation.^12^ These factors increase the risk of developing CVD. Moreover, a bidirectional Mendelian randomization study indicated that sarcopenia might causally contribute to the development of CVD,^13^ highlighting the importance of exploring this link further to identify individuals at high risk for new‐onset CVD. Moreover, there may be a bidirectional relationship between cardiovascular disease and sarcopenia, with studies suggesting that individuals at higher risk of cardiovascular disease are more likely to develop sarcopenia.^14^

Although a previous study based on data of CHARLS (China Health and Retirement Survey) has explored the link between sarcopenia and new‐onset composite CVD,^15^ it was limited by a follow‐up duration of only 3 years, which may not be sufficient to capture the long‐term cardiovascular risk related to sarcopenia. Additionally, although the relationship between handgrip strength (HGS) and CVD has been studied,^16^ the links between other baseline AWGS 2019‐defined sarcopenia components—including 6‐meter walking speed test (6‐WT), 5‐time chair stand test (5‐CST), and appendicular skeletal muscle mass (ASM)—and CVD risk remain unclear. Moreover, although AWGS 2019 does not specify sex‐specific diagnostic thresholds for 5‐CST and 6‐WT, prior research suggests that physical function may exhibit sex‐related variations due to differences in muscle mass, fat mass, and hormonal regulation.^17^ If 5‐CST and 6‐WT are found to be associated with CVD, it remains essential to explore potential sex differences in their utility for cardiovascular risk stratification.

The primary objective of our study was to assess whether sarcopenia (both definite and possible) and its components are associated with new‐onset CVD and all‐cause mortality in a large prospective cohort of middle‐aged and older Chinese adults without a history of CVD within a 5‐year follow‐up period.

## METHODS

The data utilised in this study were obtained from the CHARLS, all original data are publicly available upon approved research access from the CHARLS website (http://charls.pku.edu.cn/en). The datasets analysed, along with the analytical methods and research materials applied, are available from the corresponding author upon reasonable request.

### Data Resource

This study was derived from the CHARLS database, a nationally representative longitudinal cohort of middle‐aged and older adults established in 2011. A multistage probability sampling strategy with probability proportional to size sampling was employed to ensure the sample's representativeness, allowing for national estimates through weighting. CHARLS has received ethical approval from the Biomedical Ethics Committee of Peking University (IRB00001052‐11015), with the cohort profile detailed in previous publications.^18^ The study strictly followed the 1964 Helsinki Declaration in all procedures involving human participants. Informed written consent was obtained from all participants, ensuring full ethical compliance. In 2011, the CHARLS research team recruited 17 708 participants from approximately 10 000 households across 150 counties or districts and 450 villages in 28 provinces of China, using structured questionnaires in face‐to‐face interviews to collect data on demographics, health status, and biological markers every 2 to 3 years. More participants were included in 2015, with a total of 21 095 participants surveyed.

### Study Participants

We selected participants from CHARLS‐2015 for baseline with the following exclusion criteria: (1) missing sarcopenia assessment data; (2) failure to fully complete the HGS, 5‐CST, and 6‐WT (participants aged <60 years were not required this in the study design, therefore were considered normal 6‐WT); (3) missing age, sex, height, or weight information; (4) age<45 years; (5) presence or history of CVD at baseline; and (6) missing follow‐up data for CVD.

### Sarcopenia Assessment

Given that CHARLS is an Asian population cohort, we used the AWGS 2019 criteria to define sarcopenia, which include low muscle strength, low physical performance, and low muscle mass. HGS was measured using a Yuejian WL‐1000 dynamometer, with the highest value from 3 attempts per hand used in the analysis. Low muscle strength was defined as HGS <28.0 kg for men and <18.0 kg for women.

Physical performance was assessed via the 5‐CST and a 2.5‐meter walking test, which was converted to a 6‐WT. The Short Physical Performance Battery score required by AWGS 2019 could not be calculated due to missing data, so low physical performance was defined as 5‐CST ≥12.0 s or 6‐WT <1.0 m/s.

Muscle mass was calculated using a validated equation for Asian populations: ASM=0.193×weight (kg)+0.107×height (cm)−4.157×sex−0.037×age−2.631, where sex values were 1 for men and 2 for women, which demonstrated a high correlation with ASM measured from dual energy X‐ray absorptiometry (r=0.941) and strong predictive accuracy (R^2^=0.90, paired *t* test *P*=0.761).^19^ The calculated ASM was further adjusted for height to obtain the skeletal muscle index (SMI): SMI=ASM/height (cm)^2^. Low muscle mass was defined as SMI less than the smallest quintile of the sex‐specific study cohort,^20^ with thresholds set at SMI <6.97 kg/m^2^ for men and <5.27 kg/m^2^ for women.

Based on AWGS 2019, we classified participants into 4 groups: (1) no sarcopenia, (2) possible sarcopenia (low HGS or 5‐CST ≥12 seconds), (3) sarcopenia (low muscle mass with low HGS /or low physical performance), and (4) severe sarcopenia (low muscle mass, low HGS, and low physical performance).^7^

### CVD Diagnosis

The composite CVD included heart disease and stroke. Heart disease was identified through self‐reported doctor diagnoses: “Have you been told by a doctor that you have been diagnosed with a heart attack, angina, coronary heart disease, heart failure, or other heart problems?” Similarly, stroke was assessed by the question “Have you been told by a doctor that you have been diagnosed with a stroke?” The identification of CVD was consistent with previous studies with CHARLS.^15^, ^16^, ^21^

### Study Outcomes and Follow‐Up

Baseline data were collected in 2015, with follow‐up surveys conducted in 2018 and 2020. The primary outcome was 5‐year new‐onset composite CVD, including heart disease and stroke. The secondary outcomes were heart disease, stroke, and 5‐year all‐cause mortality (confirmed through follow‐up interviews with participants or their household members in the 2018 and 2020 waves). The interview dates for all 3 waves were recorded. Notably, in this study, due to missing time records of heart disease, stroke, and mortality for some patients, we recorded the earliest occurrence of heart disease or stroke. For follow‐up of composite CVD, the earliest occurrence of heart disease or stroke was recorded. If both composite CVD and mortality occurred in the same year, we recorded it as a CVD event. When heart disease and stroke occurred in the same year or when timing was unclear, we used separate follow‐up times for each condition in our analysis.

### Power Calculations

As this analysis was based on a data set with a fixed sample size, a post hoc power calculation was conducted using the R package ‘powerSurvEpito’ to assess whether the study was adequately powered to detect associations between sarcopenia status and incident CVD.^22^ Assuming a 2‐sided α of 0.05, a total of 1649 events, and using the no sarcopenia group as the reference, we estimated the power to detect a hazard ratio (HR) of 1.5 for each of the 3 comparison groups (possible sarcopenia, sarcopenia, and severe sarcopenia). The number of CVD events in each group was as follows: no sarcopenia (1025 events; 14.0%), possible sarcopenia (352 events; 21.7%), sarcopenia (197 events; 16.2%), and severe sarcopenia (75 events; 15.2%). The calculations showed >90% power for all group comparisons (possible sarcopenia versus no sarcopenia: 99.99%; sarcopenia versus no sarcopenia: 99.99%; severe sarcopenia versus no sarcopenia: 97.45%), indicating sufficient sample size to detect moderate associations.

### Covariates Extraction

Baseline covariates included demographic and lifestyle characteristics, comorbidities, medication use, and laboratory parameters (Table 1). Body mass index (BMI) was calculated by weight (kg)/height (m)^2^ and estimated glomerular filtration rate was calculated using the 2009 Chronic Kidney Disease Epidemiology Collaboration equation.^23^ BMI was categorized according to the World Health Organization classification criteria for the Asian population, with underweight (BMI<18.5 kg/m^2^), normal weight (18.5≤BMI<23 kg/m^2^), overweight (23≤BMI<27.5 kg/m^2^), and obesity (BMI≥27.5 kg/m^2^).^24^ Based on the study design of CHARLS, waist circumference was measured by trained researchers with the subject standing upright and after normal exhalation, the measuring tape is placed horizontally on the clothing and it is measured at the level of the navel. According to the World Health Organization and the International Diabetes Federation guidelines, abdominal obesity for Asian populations was defined as waist circumference ≥90 cm in men and ≥80 cm in women.^25^

**Table 1:** Baseline Characteristics of Patients Stratified by Sarcopenia Status

Characteristics	No sarcopenia N=7315	Possible sarcopenia N=1623	Sarcopenia N=1217	Severe sarcopenia N=494	*P* value
Age, y	69.44 (66.78–73.35)	72.63 (78.14–68.54)	73.35 (69.00–79.42)	80.65 (74.21–85.35)	<0.001
Age≥70 y, n (%)	1390 (19.0%)	757 (46.6%)	818 (67.2%)	428 (86.6%)	<0.001
Male sex, n (%)	3551 (48.5%)	705 (43.4%)	574 (47.2%)	239 (48.4%)	0.003
BMI, kg/m^2^	24.07 (22.30–26.27)	24.61 (22.87–26.67)	19.65 (18.55–20.48)	19.38 (17.54–20.63)	<0.001
BMI categories, n (%)					<0.001
Underweight	163 (2.2%)	0 (0.0%)	304 (25.0%)	164 (33.2%)	
Normal weight	2444 (33.4%)	468 (28.8%)	896 (73.6%)	315 (63.8%)	
Overweight	3465 (47.4%)	822 (50.6%)	17 (1.4%)	15 (3.0%)	
Obesity	1243 (17.0%)	333 (20.5%)	0 (0.0%)	0 (0.0%)	
Waist circumference, cm					<0.001
Abdominal obesity, n (%)	4275 (58.4%)	1127 (69.4%)	223 (18.3%)	106 (21.5%)	<0.001
Mean systolic BP, mm Hg	130.33 (118.33–144.33)	133.33 (119.67–148.00)	126.50 (113.00–142.42)	130.33 (115.17–144.17)	<0.001
Mean diastolic BP, mm Hg	75.00 (68.33–82.67)	75.00 (68.00–82.33)	71.00 (64.33–71.00)	70.67 (63.5–77.67)	<0.001
Lifestyle factors					
Smoking status, n (%)					<0.001
Never	5197 (71.0%)	1116 (68.8%)	694 (57.0%)	291 (58.9%)	
Quit	427 (5.8%)	128 (7.9%)	75 (6.2%)	51 (10.3%)	
Still smoke	1691 (23.1%)	379 (23.4%)	448 (36.8%)	152 (30.8%)	
Drinking status, n (%)					<0.001
None of these	4469 (61.1%)	1140 (70.2%)	816 (67.1%)	352 (71.3%)	
Less than once/month	684 (9.4%)	125 (7.7%)	74 (6.1%)	34 (6.9%)	
Over once/month	2162 (29.6%)	358 (22.1%)	327 (26.9%)	108 (21.9%)	
Comorbidities, n (%)					
Hypertension	1084 (14.8%)	406 (25.0%)	165 (13.6%)	90 (18.2%)	<0.001
Dyslipidemia	504 (6.9%)	132 (8.1%)	40 (3.3%)	17 (3.4%)	<0.001
Diabetes	270 (3.7%)	100 (6.2%)	41 (3.4%)	13 (2.6%)	<0.001
Malignant and cancer	56 (0.8%)	13 (0.8%)	11 (0.9%)	4 (0.8%)	0.967
Liver disease	217 (3.0%)	53 (3.3%)	36 (3.0%)	15 (3.0%)	0.936
Chronic lung disease	478 (6.5%)	181 (11.2%)	150 (12.3%)	92 (18.6%)	<0.001
Kidney disease	326 (4.5%)	94 (5.8%)	77 (6.3%)	22 (4.5%)	0.01
Medications, n (%)					
Cancer medication	71 (1.0%)	17 (1.0%)	12 (1.0%)	5 (1.0%)	0.994
Hypertension medication	1832 (25.0%)	634 (39.1%)	256 (21.0%)	127 (25.7%)	<0.001
Diabetes medication	492 (6.7%)	173 (10.7%)	55 (4.5%)	29 (5.9%)	<0.001
Sarcopenia components					
5‐time chair stand test, s	8.37 (6.99–9.81)	13.25 (12.00–15.57)	9.59 (7.85–11.93)	11.88 (9.31–16.08)	<0.001
Appendicular skeletal muscle/height, kg/m^2^	7.06 (5.97–7.64)	7.00 (5.92–7.52)	5.24 (4.86–6.68)	5.22 (4.64–6.55)	<0.001
Handgrip strength, kg	32.10 (26.15–39.00)	24.00 (18.35–29.73)	28.05 (22.00–34.00)	16.50 (13.05–23.00)	<0.001
Walking speed, m/s*	0.85 (0.71–1.00)	0.69 (0.56–0.82)	0.74 (0.61–0.86)	0.61 (0.48–0.73)	<0.001
Laboratory measurements					
White blood cell count, 10^9^/L	5.80 (4.80–6.93)	5.81 (4.80–7.00)	5.52 (4.50–6.60)	5.63 (4.60–7.16)	<0.001
Triglycerides, mg/dL	116.81 (85.84–172.12)	127.43 (91.15–185.84)	90.27 (70.80–126.55)	92.92 (70.80–135.84)	<0.001
High‐density lipoprotein cholesterol, mg/dL	49.81 (43.24–57.12)	48.65 (42.08–55.98)	55.60 (47.49–64.09)	53.28 (46.33–61.00)	<0.001
Low‐density lipoprotein cholesterol, mg/dL	103.47 (85.33–122.78)	104.25 (86.10–125.87)	101.16 (82.14–120.78)	100.00 (77.60–122.20)	0.063
Total cholesterol, mg/dL	184.17 (161.39–208.49)	185.71 (164.09–213.13)	181.27 (157.92–208.98)	179.54 (154.44–211.39)	0.059
Estimated glomerular filtration rate, mL/min per 1.73 m^2^	87.43 (82.57–91.88)	85.94 (79.65–90.82)	84.92 (78.86–90.27)	80.62 (74.65–86.73)	<0.001
Glucose, mg/dL	97.30 (90.09–106.31)	97.30 (90.09–108.11)	91.89 (86.49–100.90)	93.69 (86.49–102.70)	<0.001
Cystatin C, mg/L	0.87 (0.76–0.97)	0.91 (0.79–1.02)	0.89 (0.78–1,01)	0.95 (0.83–1.09)	<0.001
Hemoglobin A1C, %	5.90 (5.60–6.20)	5.90 (5.60–6.30)	5.80 (5.60–6.10)	5.80 (5.60–6.10)	<0.001

Data are shown as median (interquartile range), or numbers (percentages). BMI indicates body mass index; and BP, blood pressure.

* The sample size of the walking speed cohort was 5896 because patients (age≤60 years) were not asked to finish the walking test.

### Statistical Analysis

The variables extracted in this study contain different proportion of missing values, up to about 30% (Table S1), and we use ‘miceforest’ in Python to perform multiple imputation. Continuous variables were presented as mean±SD or median and interquartile range, with group differences tested using Student's *t* test, 1‐way ANOVA, Mann–Whitney *U* test, or Kruskal–Wallis H‐test. Categorical variables were presented as counts and percentages, with differences tested using chi‐square or Fisher's Exact test. After the proportional hazards tests by using the Schoenfeld residual test (Table S2), multivariable Cox proportional hazards regression analysis was performed to assess the association between sarcopenia groups or components and study outcomes, with results expressed as HR and 95% CIs. Due to the middle‐aged and older cohort in this study, all‐cause mortality events may overestimate the cumulative incidence of new‐onset composite CVD. To correct for the competing risks of all‐cause mortality for new‐onset composite CVD, we additionally used “tidycmprsk” in R for the Fine–Gray subdistribution hazard model (Fine–Gray model) with subdistribution HR (SHR) and 95% CI. Subgroup analyses were stratified by age, sex, high BMI (BMI <23 kg/m^2^ versus BMI >23 kg/m^2^), abdominal obesity, hypertension, and chronic lung disease. Restricted cubic spline analysis was used to explore the nonlinear relationship between sarcopenia components and new‐onset composite CVD. We evaluated models with different numbers of knots (ranging from 3 to 6), and selected the optimal model based on the lowest Akaike information criterion and Bayesian information criterion values. Given the relatively large sample size of our study, priority was given to Bayesian information criterion in model selection, as it imposes a stricter penalty for model complexity. Details of the selected number and placement of knots for each sarcopenia component are provided in Table S3.

For sarcopenia components significantly associated with new‐onset composite CVD, we assessed sex‐specific interaction effects and used the ‘survminer’ package in R to determine optimal cutoff points, enabling more accurate stratification. Kaplan–Meier curves were used to compare the cumulative incidence of the new‐onset composite CVD across different sarcopenia groups.

We estimated 3 Cox proportional hazards models. We selected adjusted confounders with *P*<0.05 in univariate Cox proportional hazards models for the new‐onset composite CVD (Table S4) and with clinical relevance and excluded variables with variance inflation factors >10 to avoid strong multicollinearity (Table S5). Model 1 was unadjusted; Model 2 was adjusted for age, sex, BMI, waist circumference, systolic blood pressure, diastolic blood pressure, and smoking and drinking status; Model 3 was further adjusted for comorbidities (hypertension, dyslipidemia, diabetes, chronic lung disease, kidney disease), medication use (for hypertension and diabetes), and laboratory measurements (white blood cell count, triglycerides, high‐density lipoprotein cholesterol, glucose, cystatin C, glycated hemoglobin, estimated glomerular filtration rate). Additionally, Fine–Gray models were adjusted for covariates in Model 3.

To further investigate potential reverse causality bias, we excluded individuals who developed the new‐onset composite CVD within 1‐year follow‐up to assess the association of sarcopenia status and its components with new‐onset composite CVD. Moreover, given the relatively small sample size in the severe sarcopenia group (N=494) and the limited number of events, we combined the sarcopenia and severe sarcopenia groups to increase statistical power and provide more robust estimates of the association between sarcopenia status and new‐onset composite CVD.

All analyses were performed using SPSS software (version 29.0.0.1, IBM Corporation, Armonk, NY), STATA (version 17.0, StataCorp LLC, College Station, TX), R (version 4.2.1, R Foundation for Statistical Computing, Vienna, Austria) and Python (version 3.11.4, Python Software Foundation, Beaverton, OR). Two‐tailed *P*<0.05 was considered statistically significant.

### Ethical Approval

China Health and Retirement Survey has been approved by the Biomedical Ethics Committee of Peking University, with ethical approval number: IRB00001052‐11015. For this study, it was only analyzed with anonymized data from CHARLS and therefore did not require ethic approval.

### Data Sharing

The data sets used in this study can be requested from the first author (Yang Chen) or corresponding author (Gregory Y.H. Lip).

### Transparency

The lead author (Yang Chen) affirms that the article is an honest, accurate, and transparent account of the study being reported; that no important aspects of the study have been omitted; and that any discrepancies from the study as planned have been explained.

## RESULTS

### Characteristics of Participants at Baseline

**Figure 1:**
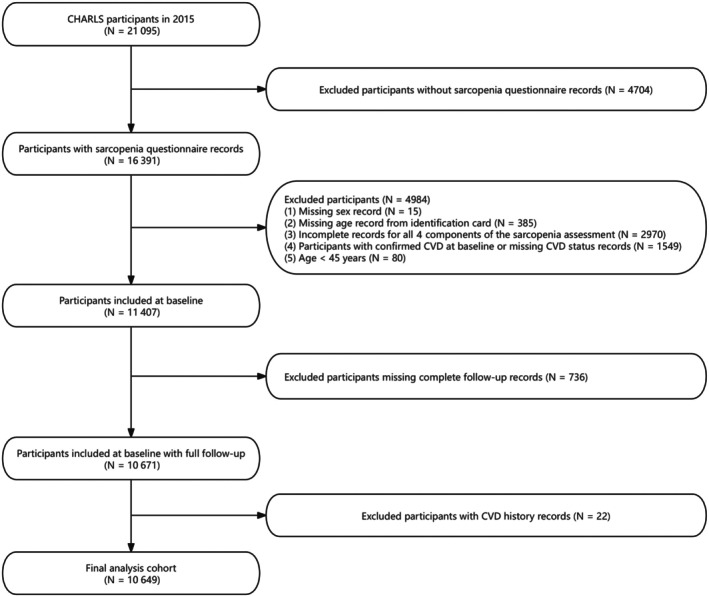
Flow chart of this study. CHARLS indicates China Health and Retirement Survey; and CVD, cardiovascular disease.

We included 10 649 participants at baseline (Figure 1), with a median age of 65.7 years (interquartile range, 55.1–72.0 years), of whom 47.6% were men. The prevalence of possible sarcopenia, sarcopenia, and severe sarcopenia was 15.2%, 11.4%, and 4.6%, respectively (Table 1). Participants with sarcopenia were generally older and had a lower BMI compared with those without sarcopenia. Individuals with severe sarcopenia represented the oldest cohort, with the lowest BMI and the highest rate of hypertension. Additionally, the CHARLS‐2015 individuals excluded from this analysis had similar age and sex proportions (median age: 63.6 years [interquartile range, 58.1–71.3 years]; 47.6% men).

### Sarcopenia Status and Outcomes

During mean follow‐up of 4.60±1.06 years, there were 661 (6.2%) all‐cause deaths and 1649 (15.5%) sustained the new‐onset CVD, including 1189 (11.2%) cases of heart disease, and 602 (5.7%) cases of stroke. Figure S1A demonstrates a strongly statistically significant difference in the cumulative risk of new‐onset composite CVD across different levels of sarcopenia (log‐rank *P*<0.001), with the highest cumulative incidence observed in participants with possible sarcopenia rather than those with no sarcopenia or severe sarcopenia. After adjusting for covariates (Model 3), neither sarcopenia nor severe sarcopenia was significantly linked to new‐onset composite CVD, heart disease, or stroke when compared with those without sarcopenia. Possible sarcopenia was significantly associated with an increased risk of the new‐onset composite CVD (HR, 1.21 [95% CI, 1.06–1.37]; stroke: HR, 1.48 [95% CI, 1.21–1.81]) (Table 2). Possible sarcopenia, sarcopenia and severe sarcopenia were significantly associated with higher all‐cause mortality compared with no sarcopenia (possible sarcopenia: HR, 1.48 [95% CI, 1.17–1.86]; sarcopenia: HR, 1.33 [95% CI, 1.01–1.76]; severe sarcopenia: HR, 1.98 [95% CI, 1.45–2.70]). Fine–Gray models, which accounted for competing risks of all‐cause mortality, supported these findings, showing that possible sarcopenia was significantly associated with both new‐onset composite CVD (SHR, 1.20 [95% CI, 1.05–1.35]) and stroke (SHR, 1.47 [95% CI, 1.20–1.80]) (Table 2). After excluding those who developed the new‐onset composite CVD within the 1‐year follow‐up, possible sarcopenia was associated with a marginal hazard effect for the development of the new‐onset composite CVD (Table S6). Moreover, after combining groups with sarcopenia and severe sarcopenia, their association with new‐onset composite CVD remained nonsignificant when compared with the group without sarcopenia (Table S7).

**Table 2:** Model Coefficients With and Without Competing Risk Adjustment for Groups Stratified by Sarcopenia Status and Outcomes

	Events (%)	Cox proportional hazards model						Fine–Gray model	
		Model 1		Model 2		Model 3		Model 3	
		HR (95% CI)	*P* value	HR (95% CI)	*P* value	HR (95% CI)	*P* value	Subdistribution HR (95% CI)	*P* value
Composite CVD	1649 (15.5)								
No sarcopenia	1025 (14.0)	Reference		Reference		Reference			
Possible sarcopenia	352 (21.7)	1.62 (1.44–1.83)	<0.001	1.24 (1.09–1.41)	0.001	1.21 (1.06–1.37)	0.005	1.20 (1.05–1.35)	0.005
Sarcopenia	197 (16.2)	1.19 (1.02–1.39)	0.024	1.03 (0.86–1.24)	0.743	1.07 (0.89–1.28)	0.489	1.06 (0.88–1.26)	0.550
Severe sarcopenia	75 (15.2)	1.15 (0.91–1.46)	0.235	0.83 (0.64–1.09)	0.175	0.85 (0.65–1.10)	0.218	0.81 (0.62–1.06)	0.130
Heart disease	1189 (11.2)								
No sarcopenia	754 (10.3)	Reference		Reference		Reference			
Possible sarcopenia	239 (14.7)	1.46 (1.26–1.69)	<0.001	1.17 (1.00–1.36)	0.052	1.13 (0.97–1.32)	0.129	1.12 (0.96–1.30)	0.140
Sarcopenia	142 (11.7)	1.16 (0.97–1.39)	0.107	1.05 (0.84–1.30)	0.688	1.08 (0.87–1.34)	0.474	1.07 (0.87–1.32)	0.520
Severe sarcopenia	54 (10.9)	1.12 (0.85–1.47)	0.430	0.88 (0.64–1.20)	0.421	0.88 (0.64–1.21)	0.431	0.85 (0.62–1.16)	0.310
Stroke	602 (5.7)								
No sarcopenia	341 (4.7)	Reference		Reference		Reference			
Possible sarcopenia	159 (9.8)	2.16 (1.79–2.61)	<0.001	1.53 (1.25–1.87)	<0.001	1.48 (1.21–1.81)	<0.001	1.47 (1.20–1.80)	<0.001
Sarcopenia	73 (6.0)	1.32 (1.02–1.70)	0.033	1.08 (0.80–1.46)	0.629	1.12 (0.83–1.52)	0.463	1.11 (0.82–1.50)	0.500
Severe sarcopenia	29 (5.9)	1.33 (0.91–1.94)	0.146	0.85 (0.55–1.30)	0.447	0.89 (0.58–1.38)	0.606	0.86 (0.55–1.32)	0.480
All‐cause mortality	610 (5.7)								
No sarcopenia	227 (3.1)	Reference		Reference		Reference			
Possible sarcopenia	125 (7.7)	2.67 (2.14–3.32)	<0.001	1.57 (1.25–1.98)	<0.001	1.48 (1.17–1.86)	0.001		
Sarcopenia	130 (10.7)	3.58 (2.88–4.44)	<0.001	1.33 (1.01–1.76)	0.042	1.33 (1.01–1.76)	0.045		
Severe sarcopenia	128 (25.9)	8.95 (7.21–11.12)	<0.001	2.14 (1.57–2.91)	<0.001	1.98 (1.45–2.70)	<0.001		

Model 1: unadjusted. Model 2: Model 1 adjusted by age, sex, body mass index, waist circumference, systolic blood pressure, diastolic blood pressure, smoking status, drinking status. Model 3: Model 2 adjusted by comorbidities (hypertension, dyslipidemia, diabetes, chronic lung disease, kidney disease), medications (hypertension medication, diabetes medication), laboratory measurements (white blood cell, triglycerides, high‐density lipoprotein cholesterol, glucose, cystatin C, hemoglobin A1C, estimated glomerular filtration rate). CVD indicates cardiovascular disease; and HR, hazard ratio.

### Subgroup Analysis for Association of Sarcopenia Status and 5‐Year Outcomes

Subgroup analyses (Tables S8 and S9) revealed significant interactions between age subgroups and sarcopenia status in relation to the new‐onset composite CVD (*P*‐for‐interaction=0.023), heart disease (*P*‐for‐interaction=0.030), and stroke (*P*‐for‐interaction=0.037). There was also a significant interaction between abdominal obesity and sarcopenia status in relation to new‐onset heart disease (*P*‐for‐interaction=0.010). No significant interactions were found in other subgroups.

### Sarcopenia Components and Outcomes

**Figure 2:**
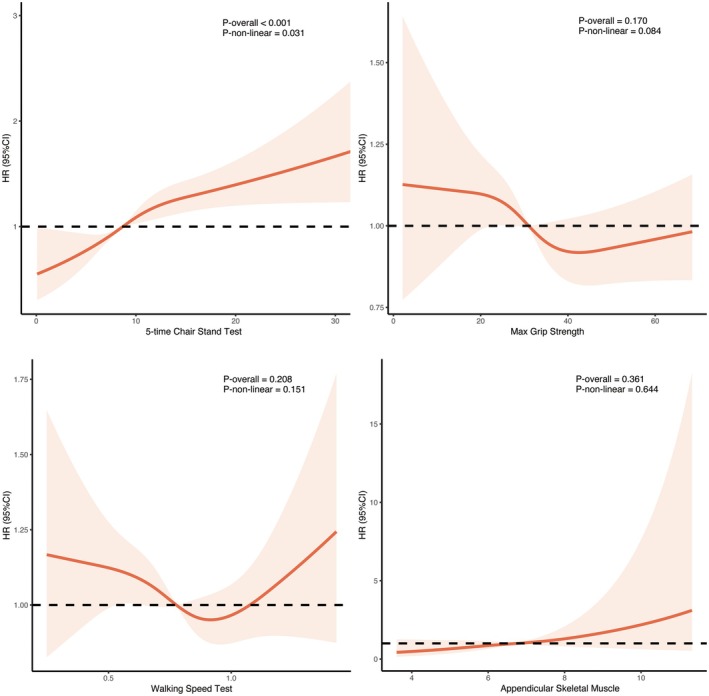
Restricted cubic spline analyses of continuous sarcopenia components and composite CVD outcome. CVD indicates cardiovascular disease; and HR, hazard ratio.

Impaired 5‐CST performance (per 1‐second increase) was linked to a higher risk of the outcome of new‐onset composite CVD (HR, 1.22 [95% CI, 1.08–1.38]), stroke (HR, 1.60 [95% CI, 1.32–1.93]), and all‐cause mortality (HR, 1.61 [95% CI, 1.34–1.93]) but not for new‐onset heart disease (Table S10). Other components such as low HGS, low 6‐WT performance, and low SMI were not associated with new‐onset composite CVD, heart disease, or stroke. The Fine–Gray model confirmed these findings (Table S11). Figure 2 illustrates the restricted cubic spline analysis results for sarcopenia components and the new‐onset composite CVD, showing that impaired 5‐CST was significantly correlated with the new‐onset composite CVD (*P*‐overall<0.001 and *P‐*nonlinear=0.031), whereas HGS, SMI, and 6‐WT were not. Therefore, 5‐CST was used for subsequent analyses.

### Sex Interaction of 5‐CST With the Composite CVD Outcome

**Figure 3:**
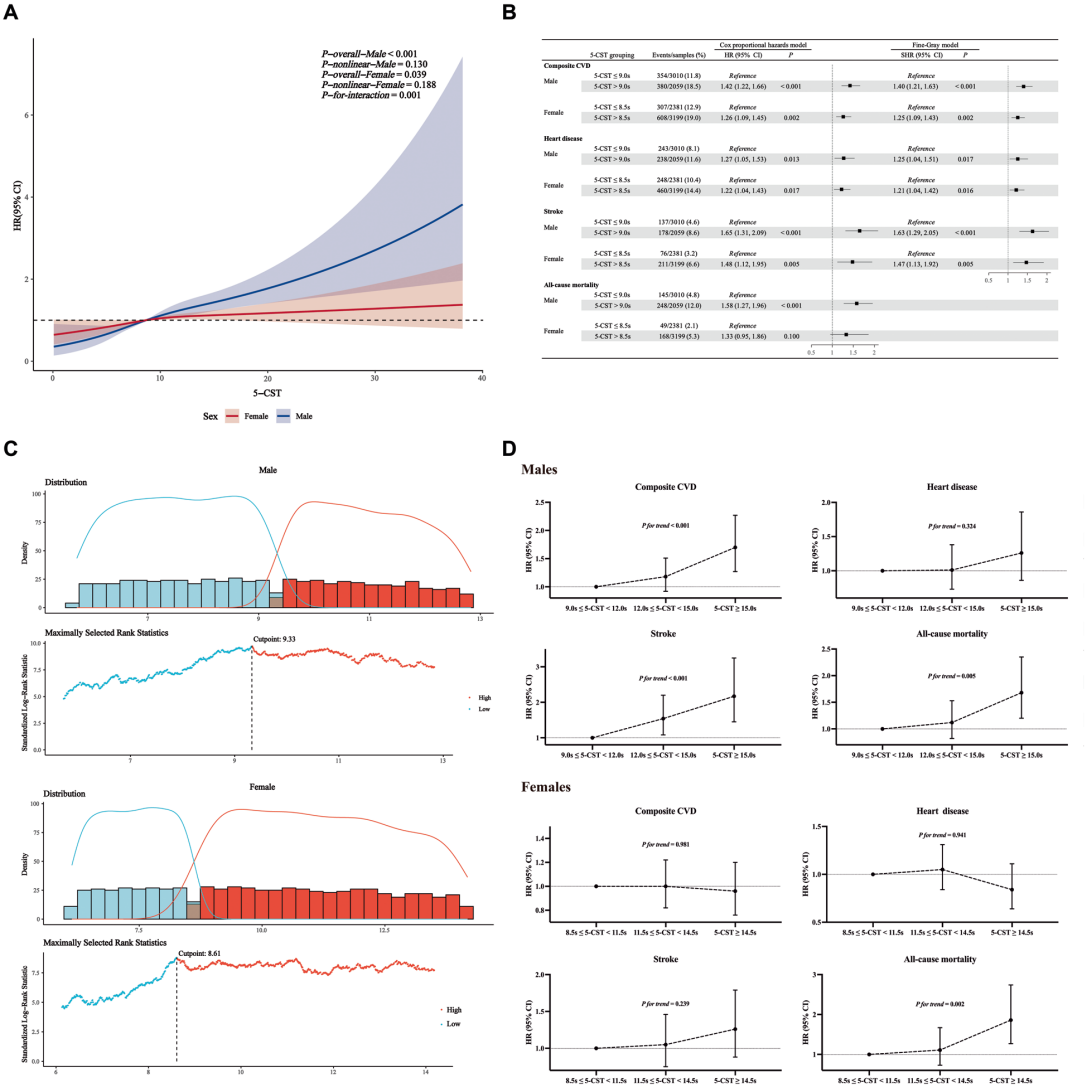
Analyses of sex interaction between 5‐CST and CVD: RCS analysis (A), optimal cutoff points (B), Cox proportional hazards model (C), and trends after the optimal cutoff points (D) Cox proportional hazards model and Fine–Gray models adjusted by age, body mass index, waist circumference, systolic blood pressure, diastolic blood pressure, smoking status, drinking status, comorbidities (hypertension, dyslipidemia, diabetes, chronic lung disease, kidney disease), medications (hypertension medication, diabetes medication), laboratory measurements (white blood cell count, triglycerides, high‐density lipoprotein cholesterol, glucose, cystatin C, hemoglobin A1C, estimated glomerular filtration rate). 5‐CST indicates 5‐time chair stand test; CVD, cardiovascular disease; HR, hazard ratio; RCS, restricted cubic spline; and SHR, subdistribution hazard ratio.

Figure 3A shows that 5‐CST is significantly associated with new‐onset composite CVD in both men (*P*‐overall<0.001) and women (*P*‐overall=0.039), without significant nonlinear relationships. There was a significant interaction between sex and 5‐CST in relation to new‐onset composite CVD (*P*‐for‐interaction=0.001). Using the maximally selected rank statistics method in the survminer package in R, we identified sex‐specific optimal cutoff points for 5‐CST as 9.3 s for men and 8.6 s for women (Figure 3B). For clinical practicality, we set the cutoff points at 9.0 s for men and 8.5 s for women.

Furthermore, our chosen thresholds effectively stratify the population: among 10 649 participants, 5290 (49.7%) had 5‐CST values above these thresholds. Approximately half of the participants (49.7%) had 5‐CST values above these thresholds, indicating that these cutoff points effectively divide the population into 2 roughly equal groups, which supports their potential applicability in clinical settings for CVD risk stratification. Multivariable Cox proportional hazards regression analysis and Fine–Gray models (Figure 3C) indicated that participants with 5‐CST above these thresholds had a higher risk of the new‐onset composite CVD, heart disease, and stroke compared with those with lower 5‐CST.

### Final Stratification Criteria of 5‐CST and Outcomes

In the restricted cubic spline analysis, the risk of the new‐onset composite CVD continued to trend higher with increasing 5‐CST after the referral point (9.0), and there were higher thresholds for 5‐CST in AWGS 2019 (12.0 s for men and women) and in European Working Group on Sarcopenia in Older People (15.0 s for men). For men, 5‐CST>9.0 s was further divided into 3 groups: 9.0 s<5‐CST≤12.0 s, 12.0 s<5‐CST≤15.0 s, and 5‐CST>15.0 s. For women, 5‐CST>8.5 s was further divided into 3 groups based on a span of 3.0 s similar to men: 8.5 s<5‐CST≤11.5 s, 11.5 s<5‐CST≤14.5 s, and 5‐CST>14.5 s. Figure 3D shows the results of the multivariable Cox proportional hazards regression analysis for men and women with 9.0 s<5‐CST≤12.0 s and 8.5 s<5‐CST≤11.5 s as the reference groups, respectively. In men, the risk of new‐onset composite CVD was significantly higher for 5‐CST > 15.0 s, whereas the difference was not significant for 12.0 s<5‐CST≤15.0 s. Consequently, the final 5‐CST cutoff points for men were set at 9.0 s and 15.0 s. It is worth noting that for new‐onset stroke, 12.0 s<5‐CST≤15.0 s also showed a significant difference. However, for women, no significant differences were found in further stratification, so the final cutoff remained at 8.5 s.

**Figure 4:**
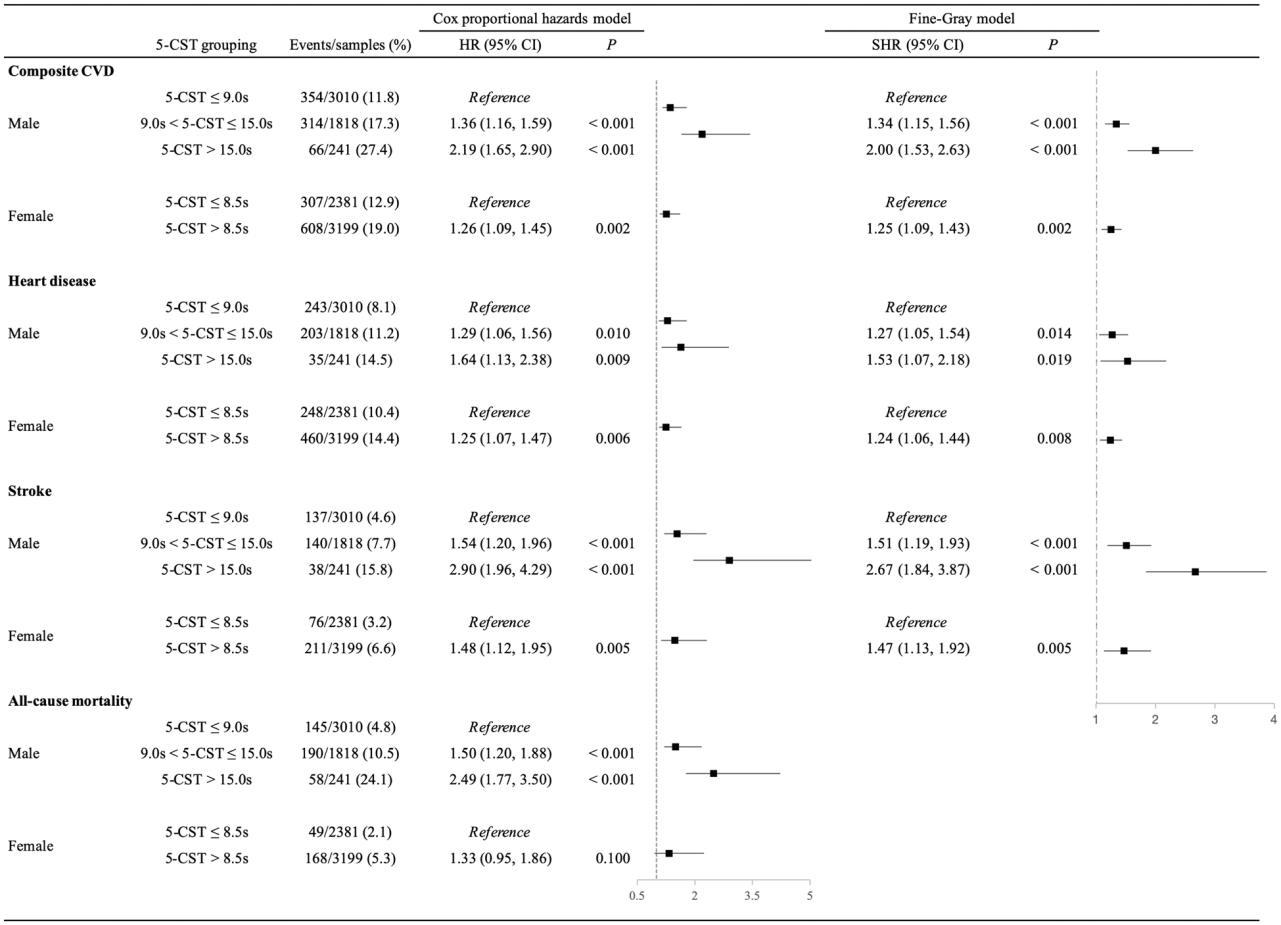
Multivariable Cox proportional hazards regression analysis and Fine–Gray models for sex‐specific newly stratified 5‐CST and outcomes. Multivariable Cox proportional hazards regression analysis and Fine–Gray models adjusted by age, body mass index, waist circumference, systolic blood pressure, diastolic blood pressure, smoking status, drinking status, comorbidities (hypertension, dyslipidemia, diabetes, chronic lung disease, kidney disease), medications (hypertension medication, diabetes medication), laboratory measurements (white blood cell count, triglycerides, high‐density lipoprotein cholesterol, glucose, cystatin C, hemoglobin A1C, estimated glomerular filtration rate). 5‐CST indicates 5‐time chair stand test; CVD, cardiovascular disease; HR, hazard ratio; and SHR, subdistribution hazard ratio.

Figure S1B and S1C demonstrate significant differences in cumulative risk of new‐onset composite CVD across 5‐CST groups in both men and women (log‐rank *P*<0.001). Figure 4 shows the results of the multivariable Cox proportional hazards regression analysis and Fine–Gray models for the final stratification criteria of 5‐CST and 5‐year outcomes. In men, compared with 5‐CST≤9.0 s, a significantly higher risk of new‐onset composite CVD was observed for both 9.0 s<5‐CST≤15.0 s (HR, 1.36 [95% CI, 1.16–1.59]; SHR, 1.34 [95% CI, 1.15–1.56]) and 5‐CST>15.0 s (HR, 2.19 [95% CI, 1.65–2.90]; SHR, 2.00 [95% CI, 1.53–2.63]). In women, 5‐CST>8.5 s was also associated with an increased risk of new‐onset composite CVD compared with 5‐CST≤8.5 s (HR, 1.26 [95% CI, 1.09–1.45]; SHR, 1.25 [95% CI, 1.09–1.43]). The sex‐specific 5‐CST thresholds remained effective for risk stratification after excluding participants who developed new‐onset composite CVD during the first year of follow‐up. As summarized in Table S12, similar associations were observed for heart disease, stroke, and all‐cause mortality.

## DISCUSSION

In this analysis of nationally surveyed middle‐aged and older adults without preexisting CVD across China, we identified several key findings: (1) sarcopenia and severe sarcopenia were significantly associated with increased all‐cause mortality but not with new‐onset composite or individual CVD; (2) possible sarcopenia was found to be linked to an elevated risk across all outcomes; and (3) for the first time, we examined the relationship between the 5‐CST and new‐onset composite CVD, identifying sex‐specific 5‐CST cutoff points for more accurate risk stratification in this population.

Several mechanisms, such as endothelial dysfunction, autonomic imbalance, chronic inflammation, insulin resistance, and impaired blood flow due to arterial stiffness, have been proposed to mediate the association between muscle strength and CVD incidence.^26^, ^27^, ^28^, ^29^, ^30^ Prior evidence on the association of sarcopenia and long‐term new‐onset CVD in middle‐aged and older populations without CVD is limited. A previous study from CHARLS by Gao et al. reported that individuals with possible sarcopenia and sarcopenia had higher risk of new‐onset composite CVD than those without sarcopenia during the 2015 to 2018 follow‐up period among participants who did not have CVD at baseline.^15^ In contrast to these prior studies, including Gao et al.'s analysis based on the CHARLS cohort, our study did not find a significant association between sarcopenia and the CVD risk. Several factors may account for this discrepancy. First, based on our analysis and evidence from other CHARLS studies,^31^, ^32^ the cross‐sectional cohort by Gao et al. had the largest sample size, yet the lowest number of sarcopenia or severe sarcopenia participants, which may affect statistical power and the generalizability of their findings. Second, our study employed a different operational definition of sarcopenia, particularly in terms of SMI cutoffs, which can substantially affect classification. Third, we had a longer follow‐up period compared with Gao et al., which may influence observed outcomes by capturing more late‐onset CVD events or competing risks such as mortality. Fourth, although we adjusted for major cardiovascular risk factors, unmeasured confounders such as nutritional intake and psychosocial factors may have influenced our results. Additionally, Shim et al. did not find an association between sarcopenia and 2‐year incidence of new‐onset atrial fibrillation in a cohort of older adults. Han et al. prospectively explored the association of sarcopenia defined by AWGS 2014 and CVD mortality in 1150 older adults (age≥60 years), finding a higher risk of CVD mortality in sarcopenia compared with no sarcopenia during a 40‐month follow‐up period.^33^ Besides, severe sarcopenia showed a nonsignificant protective effect with CVD in our study, which may be related to the limited sample size and requires a larger cohort for prospective investigation.

Our study did not find a significant association between sarcopenia and new‐onset CVD, which could be due to several factors. First, recent studies have suggested that sarcopenia often coexist with obesity, known as sarcopenic obesity, which is strongly associated with CVD, hypertension, stroke, and death.^34^ Jiang et al. found that, compared with normal weight without sarcopenia group, only overweight sarcopenia group (sum of possible sarcopenia and definite sarcopenia) was associated with the occurrence of the new‐onset composite CVD in middle‐aged and older population.^35^ However, in our cohort, the proportion of overweight individuals in definite sarcopenia or severe sarcopenia was <5.0%, whereas the proportion of overweight in possible sarcopenia was >50.0%. As overweight/obesity per se is an independent risk factor for CVD, this suggests that the combination of possible sarcopenia and overweight may drive the observed CVD risk, whereas no correlation was observed in definite or severe sarcopenia. Second, patients with possible sarcopenia in our cohort had higher levels of triglycerides and low‐density lipoprotein cholesterol, both established CVD risk factors, which may contribute to the elevated risk in this group. Third, the older age and potential comorbidities or treatment regimens (eg, antiplatelet therapy, nutritional supplements) in participants with sarcopenia and severe sarcopenia may offset their CVD risk. Furthermore, our subgroup analyses based on BMI categories did not reveal a significant interaction of BMI between sarcopenia status with CVD or mortality risk. The lack of interaction may be due to potential confounders such as age, comorbidities, or treatment regimen, apart from the relatively low prevalence of definite or severe muscle‐sparing obesity in our population. Future studies require larger sample sizes and more detailed body composition assessments to further clarify the role of sarcopenic obesity in the risk of CVD and mortality.

Possible sarcopenia demonstrated a significant link with new‐onset composite CVD, indicating that low SMI or poor 6‐WT performance may not strongly predict the occurrence of new‐onset composite CVD. Our exploratory analyses revealed that only the 5‐CST was significantly associated with new‐onset composite CVD, particularly new‐onset stroke, suggesting it could be a valuable predictor. The 5‐CST, originally designed to measure lower limb strength as part of the Short Physical Performance Battery, has been associated with CVD risk in prior studies.^36^, ^37^ Previously, Hu et al. reported that in addition to Short Physical Performance Battery, continuous 5‐CST was related to subsequent CVD and stroke in community‐dwelling older adults in the United States.^38^ However, our study has several differences: first, we detailed the sex‐specific 5‐CST cutoff points for risk stratification of new‐onset composite CVD, filling a critical research gap in the literature. Second, we excluded participants with history of CVD, allowing us to focus on the association between 5‐CST and the new‐onset composite CVD. Third, the study cohort by Hu et al.^38^ included Black and White race from the United States, whereas our cohort was Asian. Therefore, our results highlighted the potential of the 5‐CST as a simple, effective tool for assessing new‐onset CVD risk in Asian populations, though further validation in other regions is needed. Notably, our findings regarding HGS differ from those of another CHARLS analysis by Zhang et al., which reported a significant association between low HGS and increased risk of new‐onset composite CVDs.^16^ Several factors may explain this discrepancy. First, our study had a longer follow‐up period (5 years versus 3 years), which could have affected the observed associations over time. Second, our multivariable adjustment models included a wider range of cardiovascular risk factors, such as lipid indicators (triglycerides and high‐density lipoprotein cholesterol) and white blood cell count, which were not accounted for in Zhang et al.'s models.

The 5‐CST is a simple, reliable, and practical tool used to assess lower limb muscle strength and functional mobility. It requires individuals to stand up from a chair 5 times as quickly as possible, without using their arms for support, making it a potential, feasible and objective measure of muscle power in individuals without significant mobility limitations, pain, or cognitive impairment.^39^ Our study specifically aimed to establish CVD risk stratification thresholds based on 5‐CST, rather than screening sarcopenia. The thresholds identified in our analysis (9.0 s for men and 8.5 s for women) were chosen based on their ability to differentiate CVD risk, rather than aligning with current sarcopenia definitions (eg, the AWGS 2019 threshold of 12 s). Although previous literature has summarized the link between skeletal muscle decline in sarcopenia and increased CVD risk,^40^ our findings further suggest that muscle function deterioration, even before reaching the diagnostic threshold for sarcopenia, may already contribute to increased CVD risk.

Compared with HGS and 6‐WT, 5‐CST may be a more sensitive indicator of early muscle functional decline and its correlation with CVD risk. First, compared with HGS, previous studies have shown that lower limb muscle strength is more strongly associated with physical disability and functional limitations than upper limb muscle strength^41^ and that lower limb muscle mass and strength are more significantly affected by aging and are more susceptible to deterioration compared with the upper limb.^42^ Then, previous study has shown that 5‐CST is strongly correlated to 6‐WT (*r*=−0.531, *P*<0.01).^43^ However, although 6‐WT primarily reflects cardiopulmonary endurance and overall functional capacity, it is influenced by aerobic fitness, fatigue, and external environmental factors.^44^ In contrast, 5‐CST is a dynamic and time‐efficient measure, requiring only a chair and minimal space, making it more practical for large‐scale screening. However, no study was found regarding the ability of the 5‐CST and 6‐WT to accurately differentiate between lower limb muscle mass or strength, and their specific relationship to CVD risk. From our findings, 5‐CST was related with new‐onset CVD, but 6‐WT was not, further validation in larger cohorts is still needed.

The sex differences in the impact of 5‐CST on CVD risk may be linked to variations in hormone levels and muscle mass and strength. Testosterone in men has been linked to better chair stand performance, supporting muscle function and delaying functional decline,^45^ and postmenopausal estrogen loss in women accelerates muscle deterioration and physical function.^46^ Additionally, men's greater muscle mass may compensate for declining physical performance longer, explaining the higher risk thresholds (9.0 s and 15.0 s), whereas women's lower reserves lead to earlier functional impairment (>8.5 s). The precise mechanisms behind these sex differences are still unclear, necessitating further research to better understand the biological and behavioral factors involved. In addition, population‐based studies have consistently demonstrated that men have higher CVD incidence and mortality rates compared with women, with the disparity in mortality becoming more pronounced with advancing age. For example, the Global Burden of Disease Study 2021 reported that the global age‐standardized incidence and mortality rates of cardiovascular disease were higher in men (3590.60 and 1267.55 per 100 000, respectively) than in women (3161.41 and 1078.41 per 100 000, respectively), and the differences increased across older age groups.^47^ These findings underscore the importance of considering sex‐specific factors in cardiovascular risk assessment and developing targeted prevention strategies.

Our analysis shows the strong association between 5‐CST performance and the risk of new‐onset stroke. Lower extremity performance, such as walking speed, correlates with early cerebrovascular changes (eg, brain magnetic resonance imaging^48^ detected white matter changes, cerebral infarction, and ventricular enlargement) are correlated,^49^, ^50^, ^51^ and may predict ischemic stroke.^52^, ^53^ Therefore, slower walking speed may help to early identify high stroke risk. Moreover, the chair standing test may be an effective surrogate for walking speed as a measure of physical performance to screen for sarcopenia.^54^

Of note, the 5‐CST measures leg muscle strength while also reflecting sensory and balance function.^55^ Whitman et al. reported that balance and impaired gait were associated with the progressive onset of white matter disease,^56^ which was consistent with our findings. Nevertheless, our analysis did not show a significant association between the 6‐WT and the occurrence of new‐onset stroke. This may be attributed to the specific protocol used in the CHARLS cohort (2.5 meters distance rather than 6 meters). This shorter distance, although suitable for evaluating walking ability and balance, may lack the sensitivity required to adequately assess endurance and gait stability. Furthermore, these findings suggest that physical training (eg, lower limb muscle strength training, resistance training, and physical activity in general) have potential to be beneficial in preventing CVD.^57^, ^58^, ^59^

Our findings suggest that impaired 5‐CST may serve as a potential indicator of new‐onset cardiovascular risk in middle‐aged and older populations, aligning with emerging research linking sarcopenia, frailty, and CVD. Notably, our results indicate sex‐specific differences, suggesting that the association between lower limb function and cardiovascular risk may vary between men and women, highlighting the importance of sex‐stratified analyses in future studies. Although the optimal cutoff values for men and women (9.0 s and 8.5 s, respectively) are relatively close, applying a unified threshold may reduce discrimination accuracy and lead to misclassification, particularly in sex‐stratified clinical assessments. Therefore, integrating 5‐CST assessment into routine cardiovascular risk stratification has potential to help identify high‐risk individuals. Future studies should explore whether improving muscle function through resistance training or physical therapy can mitigate new‐onset cardiovascular risk and further validate the role of 5‐CST in distinguishing sarcopenia‐related muscle weakness from broader functional impairments in this population. Additionally, prospective studies are needed to compare the predictive performance of sex‐specific versus unified 5‐CST thresholds in different ethnic and clinical subgroups.

### Limitations

Our study has several limitations. First, the diagnosis of CVD in CHARLS was obtained by participant self‐reports rather than health care records. However, other cohorts have also determined the diagnosis of disease through participant self‐reported questionnaires (ie, National Health and Nutrition Examination Survey). Second, as with all observational studies, unmeasured confounders such as other anthropometric measures (ie, waist‐to‐hip ratio), nutritional intake and physical activity may have influenced our results. Third, we may have underestimated sarcopenia prevalence due to the exclusion of the Short Physical Performance Battery in our assessment. Fourth, we only considered baseline sarcopenia status, potentially overlooking the impact of new‐onset sarcopenia during the 5‐year follow‐up period. Fifth, the outcomes of follow‐up may be affected SARS‐CoV‐2, although we found no participant diagnosed SARS‐CoV‐2. The SARS‐CoV‐2 pandemic and associated restrictions in China may have influenced physical activity levels and, consequently, sarcopenia and CVD outcomes. Sixth, only Chinese participants were included in our study and so it may not be generalizable to other races or ethnicities, given the recognized ethnic difference in CVD events.^60^ Seventh, because 5‐CST performance may be influenced by conditions beyond sarcopenia, such as frailty, disability, or neurological diseases, further prospective studies are needed to isolate these factors and validate whether 5‐CST can serve as an independent indicator of new‐onset CVD. Future studies should determine whether 5‐CST distinguishes sarcopenia‐related muscle weakness from frailty, metabolic dysfunction, and obesity‐related functional decline. Eighth, although 5‐CST is used for evaluating lower limb muscle function, it may not fully reflect muscle mass loss per se, highlighting the need for extra studies to assess the relationship between 5‐CST and lower limb muscle mass. Ninth, the use equation for ASM may introduce some measurement error compared with dual energy X‐ray absorptiometry, although our findings suggest no independent association between muscle mass and new‐onset CVD, future studies using direct measurements such as dual energy X‐ray absorptiometry or bioelectrical impedance analysis are needed for confirmation. Tenth, around one third of participants were excluded due to missing components of sarcopenia assessment. Although the age and sex distribution of excluded individuals was similar to the included cohort, we cannot exclude the possibility of selection bias, which may affect generalizability. Finally, the precision of follow‐up time records may have affected our results, highlighting the need for more rigorous prospective studies.

## CONCLUSIONS

Sarcopenia, as defined by the AWGS 2019 criteria, was significantly associated with all‐cause mortality but not with new‐onset CVD in middle‐aged and older Chinese adults without prior CVD. In contrast, individuals with possible sarcopenia showed an increased risk for both new‐onset CVD and all‐cause mortality. It may suggest that the progression from possible to definite sarcopenia does not consistently correspond to an increased cardiovascular risk. Among the components of sarcopenia, the 5‐CST was linked to new‐onset composite CVD outcome, especially stroke. Sex‐specific cutoff points for the 5‐CST may provide a more precise stratification of cardiovascular risk in middle‐aged and older populations.

## Sources of Funding

None.

## Disclosures

None.

## Supporting information

Tables S1–S12
Figure S1
