# Supplementary material for: Associations of Sarcopenia and Its Components With Cardiovascular Risk: Five‐Year Longitudinal Evidence From China Health and Retirement Longitudinal Study
Source: J Am Heart Assoc. 2025 Jun 18;14(13):e040099. doi: 10.1161/JAHA.124.040099 (PMC12449978; doi:10.1161/JAHA.124.040099)
Supplement: Supplementary file 1 — Tables S1–S12 Figure S1 [file JAH3-14-e040099-s001.pdf]

Supplemental Materials

Table S1 Proportion of missing value.

Variables	Number (%)
Waist circumference	11 (0.10)
Mean systolic blood pressure	77 (0.72)
Mean diastolic blood pressure	77 (0.72)
White blood cell	2044 (19.19)
Triglycerides	1959 (18.40)
High-density lipoprotein cholesterol	1959 (18.40)
Low-density lipoprotein cholesterol	1959 (18.40)
Total cholesterol	1960 (18.41)
Estimated glomerular filtration rate	1962 (18.42)
Glucose	3226 (30.29)
Cystatin C	1964 (18.44)
Haemoglobin A1C	1925 (18.08)

Table S2 Proportional hazards assumption tests for key variables in the multivariable cox models for each outcome.

Variables	Outcomes	P value
Sarcopenia status	Composite CVD	0.960
	Heart disease	0.093
	Stroke	0.440
	All-cause mortality	0.100
Handgrip strength	Composite CVD	0.710
	Heart disease	0.091
	Stroke	0.360
	All-cause mortality	0.051
5-CST	Composite CVD	0.062
	Heart disease	0.470
	Stroke	0.097
	All-cause mortality	0.890
6-Meter walking speed test	Composite CVD	0.870
	Heart disease	0.750
	Stroke	0.970
	All-cause mortality	0.180
Skeletal muscle index	Composite CVD	0.230
	Heart disease	0.270
	Stroke	0.390
	All-cause mortality	0.260
Male’s new stratification of 5-CST	Composite CVD	0.700
	Heart disease	0.062
	Stroke	0.850
	All-cause mortality	0.690
Female’s new stratification of 5-CST	Composite CVD	0.130
	Heart disease	0.065
	Stroke	0.370
	All-cause mortality	0.056

Abbreviation: CVD, cardiovascular disease; 5-CST, 5-time chair stand test.

Table S3. Knots selection for restricted cubic spline models of sarcopenia components and new-onset composite cardiovascular disease

Outcome	Number of knots	Akaike information criterion	Bayesian information criterion	Final selected number of knots
Handgrip strength	3	29850.65	29985.85	3
	4	29850.58	29991.19	
	5	29850.15	29996.16	
	6	29850.41	30001.84	
5-Time chair stand test	3	29816.15	29951.34	3
	4	29818.38	29958.99	
	5	29817.82	29963.83	
	6	29819.34	29970.77	
6-Meter walking speed test	3	19382.48	19508.52	3
	4	19384.20	19515.28	
	5	19385.44	19521.55	
	6	19387.66	19528.82	
Skeletal muscle index	3	29853.68	29988.88	3
	4	29854.03	29994.63	
	5	29850.68	29996.69	
	6	29851.64	30003.06	

Table S4 Results of univariate Cox proportional hazards analysis for CVD.

Variable	HR	95% CI	*P*
Age	1.027	1.022-1.031	< 0.001
BMI	1.034	1.022-1.045	< 0.001
Female	1.126	1.022-1.241	0.017
Waist Circumference	1.013	1.008-1.017	< 0.001
Mean SBP	1.015	1.013-1.017	< 0.001
Mean DBP	1.013	1.009-1.017	< 0.001
Smoking Status			0.007
Never		*Reference*	
Quit	1.267	1.057-1.520	0.010
Still Smoke	0.920	0.819-1.034	0.161
Drinking Status			< 0.001
None of These		*Reference*	
Drink but less than Once a Month	0.887	0.743-1.060	0.193
Drink over Once a Month	0.795	0.709-0.892	< 0.001
Hypertension	2.031	1.822-2.264	< 0.001
Dyslipidemia	2.001	1.721-2.327	< 0.001
Diabetes	1.815	1.496-2.202	< 0.001
Malignant & Cancer	1.054	0.623-1.784	0.844
Liver Disease	1.279	0.993-1.649	0.057
Chronic Lung Disease	1.560	1.346-1.808	< 0.001
Kidney Disease	1.239	1.009-1.521	0.040
Cancer Medication	1.047	0.649-1.688	0.852
Hypertension Medication	2.111	1.915-2.328	< 0.001
Diabetes Medication	1.841	1.584-2.139	< 0.001
WBC	1.053	1.028-1.078	< 0.001
Triglycerides	1.001	1.000-1.001	< 0.001
HDL-C	0.994	0.990-0.999	0.010
LDL-C	1.002	1.000-1.003	0.013
Total Cholesterol	1.002	1.001-1.003	0.003
Glucose	1.004	1.002-1.005	< 0.001
Cystatin C	1.792	1.494-2.150	< 0.001
CRP	1.006	0.999-1.013	0.098
Hemoglobin A1C	1.170	1.128-1.214	< 0.001
eGFR	0.983	0.979-0.987	< 0.001

Abbreviation: BMI, body mass index; SBP, systolic blood pressure; CI, confidence interval; CVD, cardiovascular disease; DBP, diastolic blood pressure; eGFR, estimated glomerular filtration rate; LDL-C, low-density lipoprotein cholesterol; HDL-C, high-density lipoprotein cholesterol; HR, hazard ratio; WBC, white blood cell count; CRP, C-reactive protein.

Table S5 Results of the multi-collinearity test.

Variable	Variance Inflation Factor
Age	3.365
BMI	2.070
Female	1.915
WC	1.717
Mean SBP	2.272
Mean DBP	2.045
Smoking Status	1.589
Drinking Status	1.357
Hypertension	1.768
Dyslipidemia	1.130
Diabetes	1.537
Chronic Lung Disease	1.045
Kidney Disease	1.019
Hypertension Medication	1.862
Diabetes Medication	1.685
WBC	1.137
Triglycerides	4.505
HDL-C	2.923
LDL-C	15.607
Total Cholesterol	20.601
Glucose	2.632
Cystatin C	1.940
Hemoglobin A1C	2.881
eGFR	3.460
Sarcopenia status	1.662

Abbreviation: BMI, body mass index; SBP, systolic blood pressure; DBP, diastolic blood pressure; eGFR, estimated glomerular filtration rate; LDL-C, low-density lipoprotein cholesterol; HDL-C, high-density lipoprotein cholesterol; WBC, white blood cell count.

Table S6 Associations of sarcopenia status and new-onset CVD (excluding CVD events whith 1-year follow-up).

Sarcopenia status	Cox proportional hazards model		Fine-Gray model	
	HR (95% CI)	*P*	SHR (95% CI)	*P*
No sarcopenia	*Reference*		*Reference*	
Possible sarcopenia	1.16 (1.00, 1.35)	0.056	1.14 (0.98, 1.31)	0.080
Sarcopenia	1.00 (0.81, 1.24)	0.967	1.00 (0.81, 1.23)	0.980
Severe sarcopenia	0.77 (0.56, 1.06)	0.108	0.74 (0.54, 1.02)	0.069

Cox proportional hazards model and Fine & Gray models adjusted by age, sex, body mass index, waist circumference, systolic blood pressure, diastolic blood pressure, smoking status, drinking status, comobidities (hypertension, dyslipidemia, diabetes, chronic lung disease, kidney disease), medications (hypertension medication, diabetes medication), laboratory measurements (white blood cell, triglycerides, high-density lipoprotein cholesterol, glucose, cystatin C, hemoglobin A1C, estimated glomerular filtration rate).

Abbreviation: CI, confidence interval; CVD, cardiovascular disease; Fine-Gray model, Fine-Gray subdistribution hazard model; HR, hazard ratio; SHR, sub-distribution hazard ratio.

Table S7 Associations of sarcopenia status and new-onset CVD after combining sarcopenia and severe sarcopenia groups.

	Cox proportional hazards model		Fine-Gray model	
Sarcopenia status	HR (95% CI)	*P*	SHR (95% CI)	*P*
No sarcopenia	*Reference*		*Reference*	
Possible sarcopenia	1.22 (1.07, 1.39)	0.002	1.20 (1.06, 1.36)	0.004
Sarcopenia + Severe sarcopenia	1.00 (0.84, 1.19)	0.987	0.99 (0.83, 1.17)	0.870

Cox proportional hazards model and Fine & Gray models adjusted by age, sex, body mass index, waist circumference, systolic blood pressure, diastolic blood pressure, smoking status, drinking status, comobidities (hypertension, dyslipidemia, diabetes, chronic lung disease, kidney disease), medications (hypertension medication, diabetes medication), laboratory measurements (white blood cell, triglycerides, high-density lipoprotein cholesterol, glucose, cystatin C, hemoglobin A1C, estimated glomerular filtration rate).

Abbreviation: CI, confidence interval; CVD, cardiovascular disease; Fine-Gray model, Fine-Gray subdistribution hazard model; HR, hazard ratio; SHR, sub-distribution hazard ratio.

Table S8 Subgroup analyses for the association of sarcopenia status and cardiovascular outcomes.

	Cardiovascular Disease			Heart Disease			Stroke		
**Subgroups**	HR (95% CI)	*P*	*P for Interaction*	HR (95% CI)	*P*	*P for Interaction*	HR (95% CI)	*P*	*P for Interaction*
**Age <70 years**			0.023			0.030			0.037
Non-sarcopenia	*Reference*			*Reference*			*Reference*		
Possible sarcopenia	1.16 (0.97, 1.39)	0.105		1.02 (0.82, 1.26)	0.876		1.55 (1.17, 2.06)	0.002	
Sarcopenia	0.94 (0.69, 1.29)	0.694		0.84 (0.57, 1.23)	0.363		1.37 (0.84, 2.24)	0.207	
Severe sarcopenia	1.61 (0.94, 2.73)	0.081		1.68 (0.92, 3.04)	0.090		1.98 (0.84, 4.66)	0.120	
**Age ≥70 years**									
Non-sarcopenia	*Reference*			*Reference*			*Reference*		
Possible sarcopenia	1.28 (1.06, 1.55)	0.010		1.28 (1.02, 1.61)	0.031		1.43 (1.07, 1.90)	0.014	
Sarcopenia	1.10 (0.86, 1.40)	0.467		1.26 (0.94, 1.68)	0.123		0.91 (0.61, 1.35)	0.639	
Severe sarcopenia	0.85 (0.61, 1.18)	0.337		0.96 (0.65, 1.41)	0.817		0.79 (0.47, 1.34)	0.390	
**Male**			0.400			0.151			0.733
Non-sarcopenia	*Reference*			*Reference*			*Reference*		
Possible sarcopenia	1.40 (1.15, 1.70)	< 0.001		1.22 (0.95, 1.58)	0.117		1.63 (1.23, 2.17)	< 0.001	
Sarcopenia	1.27 (0.97, 1.67)	0.086		1.44 (1.34, 2.01)	0.031		1.14 (0.74, 1.77)	0.550	
Severe sarcopenia	1.05 (0.71, 1.54)	0.827		1.24 (0.78, 1.96)	0.369		0.90 (0.48, 1.68)	0.735	
**Female**									
Non-sarcopenia	*Reference*			*Reference*			*Reference*		
Possible sarcopenia	1.07 (0.91, 1.28)	0.412		1.07 (0.88, 1.30)	0.520		1.32 (0.99, 1.77)	0.058	
Sarcopenia	0.92 (0.72, 1.19)	0.534		0.88 (0.66, 1.17)	0.381		1.08 (0.70, 1.66)	0.735	
Severe sarcopenia	0.71 (0.49, 1.02)	0.064		0.69 (0.45, 1.06)	0.087		0.85 (0.47, 1.56)	0.608	
**No Abdominal Obesity**									
Non-sarcopenia	*Reference*			*Reference*			*Reference*		

Possible sarcopenia	1.38 (1.08, 1.76)	0.009		1.31 (0.97, 1.77)	0.075		1.72 (1.19, 2.49)	0.004	
Sarcopenia	1.22 (0.95, 1.56)	0.113		1.24 (0.93, 1.66)	0.144		1.35 (0.90, 2.03)	0.141	
Severe sarcopenia	0.97 (0.68, 1.37)	0.844		0.89 (0.59, 1.36)	0.594		1.29 (0.74, 2.24)	0.369	
**Abdominal obesity**			0.145			0.010			0.896
Non-sarcopenia	*Reference*			*Reference*			*Reference*		
Possible sarcopenia	1.11 (0.96, 1.30)	0.170		1.04 (0.87, 1.25)	0.647		1.33 (1.04, 1.70)	0.024	
Sarcopenia	0.77 (0.53, 1.11)	0.161		0.58 (0.36, 0.93)	0.024		1.10 (0.64, 1.90)	0.727	
Severe sarcopenia	0.68 (0.41, 1.13)	0.133		0.79 (0.45, 1.39)	0.416		0.67 (0.28, 1.58)	0.359	
**Low BMI**									
Non-sarcopenia	*Reference*			*Reference*			*Reference*		
Possible sarcopenia	1.52 (1.19, 1.95)	< 0.001		1.33 (0.98, 1.80)	0.067		2.09 (1.42, 3.07)	< 0.001	
Sarcopenia	1.17 (0.94, 1.46)	0.165		1.15 (0.89, 1.50)	0.287		1.39 (0.95, 2.03)	0.087	
Severe sarcopenia	0.91 (0.66, 1.25)	0.558	0.109	0.91 (0.63, 1.33)	0.632	0.408	1.12 (0.66, 1.89)	0.672	0.072
**High BMI**									
Non-sarcopenia	*Reference*			*Reference*			*Reference*		
Possible sarcopenia	1.11 (0.95, 1.29)	0.200		1.06 (0.89, 1.27)	0.503		1.28 (1.01, 1.63)	0.044	
Sarcopenia	0.80 (0.25, 2.50)	0.694		0.35 (0.05, 2.51)	0.296		1.48 (0.36, 6.10)	0.591	
Severe sarcopenia	1.34 (0.55, 3.29)	0.520		1.07 (0.34, 3.38)	0.913		2.13 (0.66, 6.91)	0.207	
**No Hypertension**									
Non-sarcopenia	*Reference*			*Reference*			*Reference*		
Possible sarcopenia	1.15 (0.98, 1.35)	0.081		1.08 (0.89, 1.30)	0.434		1.39 (1.07, 1.81)	0.015	
Sarcopenia	1.16 (0.95, 1.43)	0.156	0.123	1.10 (0.86, 1.40)	0.450	0.881	1.27 (0.89, 1.80)	0.187	0.353
Severe sarcopenia	0.85 (0.63, 1.16)	0.317		0.86 (0.60, 1.23)	0.399		0.89 (0.53, 1.49)	0.648	
**Hypertension**									
Non-sarcopenia	*Reference*			*Reference*			*Reference*		
Possible sarcopenia	1.30 (1.04, 1.62)	0.019		1.26 (0.96, 1.65)	0.099		1.57 (1.14, 2.16)	0.006	

Sarcopenia	0.68 (0.44, 1.05)	0.083		0.94 (0.57, 1.55)	0.807		0.66 (0.34, 1.29)	0.222	
Severe sarcopenia	0.75 (0.44, 1.29)	0.302		0.86 (0.44, 1.67)	0.658		0.87 (0.40, 1.90)	0.722	
**No Chronic Lung Disease**									
Non-sarcopenia	*Reference*			*Reference*			*Reference*		
Possible sarcopenia	1.18 (1.03, 1.36)	0.017		1.11 (0.94, 1.31)	0.233		1.47 (1.19, 1.82)	< 0.001	
Sarcopenia	1.07 (0.88, 1.31)	0.489		1.09 (0.86, 1.39)	0.456		1.09 (0.78, 1.51)	0.625	
Severe sarcopenia	0.83 (0.72, 1.13)	0.239		0.85 (0.59, 1.23)	0.390		0.93 (0.58, 1.48)	0.753	
			0.942			0.795			0.736
**Chronic Lung Disease**									
Non-sarcopenia	*Reference*			*Reference*			*Reference*		
Possible sarcopenia	1.47 (1.03, 2.10)	0.034		1.32 (0.87, 1.99)	0.188		1.68 (0.90, 3.14)	0.106	
Sarcopenia	1.05 (0.64, 1.72)	0.843		1.03 (0.59, 1.79)	0.928		1.39 (0.58, 3.36)	0.463	
Severe sarcopenia	0.93 (0.51, 1.70)	0.819		0.98 (0.50, 1.92)	0.959		0.78 (0.23, 2.64)	0.690	

Adjusted by age, sex, BMI, waist circumference, systolic blood pressure, diastolic blood pressure, smoking status, drinking status, comobidities (hypertension, dyslipidemia, diabetes, chronic lung disease, kidney disease), medications (hypertension medication, diabetes medication), laboratory measurements (white blood cell, triglycerides, high-density lipoprotein cholesterol, glucose, cystatin C, hemoglobin A1C, estimated glomerular filtration rate).

Abbreviation: BMI, body mass index; CI, confidence interval; HR, hazard ratio.

Table S9 Subgroup analyses for the association of sarcopenia status and all-cause mortality.

	All-cause Mortality		
**Subgroups**	HR (95% CI)	*P*	*Interaction P*
**Age <70 years**			
Non-sarcopenia	*Reference*		
Possible sarcopenia	1.76 (1.20, 2.60)	0.004	
Sarcopenia	1.46 (0.87, 2.45)	0.158	
Severe sarcopenia	3.17 (1.39, 7.23)	0.006	0.510
**Age ≥70 years**			
Non-sarcopenia	*Reference*		
Possible sarcopenia	1.30 (0.97, 1.74)	0.075	
Sarcopenia	1.18 (0.84, 1.66)	0.330	
Severe sarcopenia	1.79 (1.24, 2.57)	0.002	
**Male**			
Non-sarcopenia	*Reference*		
Possible sarcopenia	1.46 (1.09, 1.96)	0.011	
Sarcopenia	1.21 (0.86, 1.71)	0.280	
Severe sarcopenia	1.66 (1.12, 2.46)	0.011	0.469
**Female**			
Non-sarcopenia	*Reference*		
Possible sarcopenia	1.51 (1.02, 2.25)	0.040	
Sarcopenia	1.46 (0.91, 2.35)	0.117	
Severe sarcopenia	2.46 (1.45, 4.15)	< 0.001	
**No Abdominal Obesity**			
Non-sarcopenia	*Reference*		
Possible sarcopenia	1.36 (0.95, 1.95)	0.089	
Sarcopenia	1.17 (0.84, 1.64)	0.347	
Severe sarcopenia	1.71 (1.15, 2.52)	0.007	0.696
**Abdominal obesity**			
Non-sarcopenia	*Reference*		
Possible sarcopenia	1.58 (1.15, 2.18)	0.005	
Sarcopenia	1.43 (0.79, 2.57)	0.239	
Severe sarcopenia	2.37 (1.32, 4.27)	0.004	
**Low BMI**			
Non-sarcopenia	*Reference*		
Possible sarcopenia	1.39 (0.95, 2.05)	0.094	
Sarcopenia	1.35 (1.00, 1.82)	0.052	
Severe sarcopenia	2.10 (1.49, 2.97)	< 0.001	
**High BMI**			0.346
Non-sarcopenia	*Reference*		

Possible sarcopenia	1.50 (1.11, 2.03)	0.009	
Sarcopenia	2.99 (1.03, 8.71)	0.044	
Severe sarcopenia	3.17 (0.95, 10.60)	0.060	
**No Hypertension**			
Non-sarcopenia	*Reference*		
Possible sarcopenia	1.52 (1.15, 1.99)	0.003	
Sarcopenia	1.19 (0.87, 1.63)	0.270	
Severe sarcopenia	1.75 (1.23, 2.49)	0.002	0.870
**Hypertension**			
Non-sarcopenia	*Reference*		
Possible sarcopenia	1.37 (0.88, 2.15)	0.168	
Sarcopenia	1.88 (1.01, 3.49)	0.047	
Severe sarcopenia	3.05 (1.57, 5.94)	0.001	
**No Chronic Lung Disease**			
Non-sarcopenia	*Reference*		
Possible sarcopenia	1.48 (1.15, 1.91)	0.002	
Sarcopenia	1.31 (0.97, 1.78)	0.081	
Severe sarcopenia	1.91 (1.35, 2.71)	< 0.001	0.564
**Chronic Lung Disease**			
Non-sarcopenia	*Reference*		
Possible sarcopenia	1.34 (0.71, 2.53)	0.373	
Sarcopenia	1.42 (0.70, 2.91)	0.334	
Severe sarcopenia	2.15 (1.00, 4.61)	0.051	

Adjusted by age, sex, BMI, waist circumference, systolic blood pressure, diastolic blood pressure, smoking status, drinking status, comobidities (hypertension, dyslipidemia, diabetes, chronic lung disease, kidney disease), medications (hypertension medication, diabetes medication), laboratory measurements (white blood cell, triglycerides, high-density lipoprotein cholesterol, glucose, cystatin C, hemoglobin A1C, estimated glomerular filtration rate).
Abbreviation: BMI, body mass index; CI, confidence interval; HR, hazard ratio.

Table S10 Multivariate cox proportional hazard regression analysis for sarcopenia components and outcomes

	Cardiovascular Disease		Heart Disease		Stroke		All-cause Mortality	
	HR (95% CI)	*P*	HR (95% CI)	*P*	HR (95% CI)	*P*	HR (95% CI)	*P*
**Handgrip Strength, kg**								
Continuous	1.00 (0.99, 1.01)	0.822	1.00 (1.00, 1.01)	0.375	0.99 (0.97, 1.00)	0.016	0.99 (0.97, 1.00)	0.011
Categorised								
Normal handgrip strength	Reference		Reference		Reference		Reference	
Abnormal handgrip strength	1.01 (0.87, 1.17)	0.952	0.97 (0.81, 1.16)	0.726	1.07 (0.85, 1.35)	0.575	1.39 (1.14, 1.69)	< 0.001
**[a]ASM/Height^2, kg/m^2**								
Continuous	1.29 (0.92, 1.81)	0.145	1.45 (0.98, 2.16)	0.065	0.84 (0.48, 1.47)	0.542	0.45 (0.27, 0.76)	0.003
Categorised								
Normal **ASM/Height^2**	Reference		Reference		Reference		Reference	
Abnormal **ASM/Height^2**	0.94 (0.80, 1.11)	0.485	1.00 (0.83, 1.20)	0.973	0.83 (0.64, 1.09)	0.183	1.18 (0.92, 1.52)	0.195
**5-CST, s**								
Continuous	1.03 (1.02, 1.04)	< 0.001	1.02 (1.01, 1.04)	0.002	1.05 (1.04, 1.07)	< 0.001	1.04 (1.03, 1.06)	< 0.001
Categorised								
Normal 5-CST	Reference		Reference		Reference		Reference	
Abnormal 5-CST	1.22 (1.08, 1.38)	0.001	1.09 (0.94, 1.26)	0.281	1.60 (1.32, 1.93)	< 0.001	1.61 (1.34, 1.93)	< 0.001
**[b]6-WT, m/s**								
Continuous	1.01 (1.00, 1.01)	0.027	1.00 (0.99, 1.01)	0.814	1.01 (1.00, 1.02)	0.021	0.99 (0.98, 1.03)	0.498
Categorised								
Normal 6-WT	Reference		Reference		Reference		Reference	
Abnormal 6-WT	1.04 (0.88, 1.24)	0.612	1.08 (0.88, 1.32)	0.464	1.08 (0.83, 1.41)	0.574	1.56 (1.13, 2.14)	0.006

[a]ASM/Height^2 was defined as less than the lowest sex-specific 20% of the study population.

[b]The sample size of walking speed cohort was 5896 because patients (age ≤60 years) were not asked to finish walking test.

Categorised sarcopenia components were based on the criteria of AWGS2019, including abnormal handgrip strength: <28 kg for males, <18 kg for females; abnormal **ASM/Height^2**: <6.97 kg/m² for males and <5.27 kg/m² for females; abnormal 5-CST: 5-CST ≥12 s; abnormal 6-WT: <1 m/s.

Adjusted by age, sex, body mass index, waist circumference, systolic blood pressure, diastolic blood pressure, smoking status, drinking status, comobidities (hypertension, dyslipidemia, diabetes, chronic lung disease, kidney disease), medications (hypertension medication, diabetes medication), laboratory measurements (white blood cell, triglycerides, high-density lipoprotein cholesterol, glucose, cystatin C, hemoglobin A1C, estimated glomerular filtration rate).

Abbreviation: CI, confidence interval; 5-CST, 5-time chair stand test; HR, hazard ratio; 6-WT, 6-meter walking speed test.

Table S11 Multivariate Fine-Gray subdistribution hazard model for sarcopenia components and outcomes.

	Cardiovascular Disease		Heart Disease		Stroke	
	SHR (95% CI)	*P*	SHR (95% CI)	*P*	SHR (95% CI)	*P*
**Handgrip Strength**						
Continuous	1.00 (0.99, 1.00)	0.610	1.00 (0.99, 1.01)	0.780	1.00 (0.99, 1.00)	0.380
Categorised						
Normal handgrip strength	Reference		Reference		Reference	
Abnormal handgrip strength	0.99 (0.85, 1.15)	0.880	0.95 (0.80, 0.14)	0.600	1.06 (0.83, 1.34)	0.650
[a]**ASM/Height^2**						
Continuous	0.97 (0.90, 1.04)	0.410	0.90 (0.83, 0.98)	0.017	1.14 (1.01, 1.28)	0.034
Categorised						
Normal **ASM/Height^2**	Reference		Reference		Reference	
Abnormal **ASM/Height^2**	0.94 (0.80, 1.10)	0.420	1.00 (0.84, 1.20)	0.960	0.80 (0.61, 1.04)	0.100
**5-CST**						
Continuous	1.03 (1.02, 1.04)	< 0.001	1.02 (1.01, 1.04)	< 0.001	1.05 (1.03, 1.06)	< 0.001
Categorised						
Normal 5-CST	Reference		Reference		Reference	
Abnormal 5-CST	1.21 (1.07, 1.36)	0.002	1.09 (0.94, 1.25)	0.250	1.54 (1.27, 1.86)	< 0.001
[b]**Walking Speed Test**						
Continuous	1.01 (1.00, 1.01)	0.011	1.00 (0.99, 1.01)	0.830	1.01 (1.00, 1.02)	0.009
Categorised						
Normal 6-WT	Reference		Reference		Reference	
Abnormal 6-WT	1.04 (0.88, 1.22)	0.660	1.08 (0.89, 1.31)	0.430	1.05 (0.81, 1.37)	0.700

[a]ASM/Height^2 was defined as less than the lowest sex-specific 20% of the study population.

[b]The sample size of walking speed cohort was 5896 because patients (age ≤60 years) were not asked to finish walking test.

Categorised sarcopenia components were based on the criteria of AWGS2019, including abnormal handgrip strength: <28 kg for males, <18 kg for females; abnormal **ASM/Height^2**: <6.97 kg/m² for males and <5.27 kg/m² for females; abnormal 5-CST: 5-CST ≥12 s; abnormal 6-WT: <1 m/s.

Adjusted by age, sex, body mass index, waist circumference, systolic blood pressure, diastolic blood pressure, smoking status, drinking status, comobidities (hypertension, dyslipidemia, diabetes, chronic lung disease, kidney disease), medications (hypertension medication, diabetes medication), laboratory measurements (white blood cell, triglycerides, high-density lipoprotein cholesterol, glucose, cystatin C, hemoglobin A1C, estimated glomerular filtration rate).

Abbreviation: CI, confidence interval; SHR, subdistribution hazard ratio.

Table S12 Associations of new 5-CST intervals and new-onset CVD (excluding CVD events whith 1-year follow-up).

		Cox proportional hazards model		Fine-Gray model	
	5-CST grouping	HR (95% CI)	*P*	SHR (95% CI)	*P*
Male	5-CST ≤ 9.0s	*Reference*		*Reference*	
	9.0s < 5-CST ≤ 15.0s	1.30 (1.08, 1.56)	0.006	1.27 (1.06, 1.52)	0.010
	5-CST > 15.0s	1.81 (1.26, 2.58)	0.001	1.63 (1.15, 2.29)	0.006
Female	5-CST ≤ 8.5s	*Reference*		*Reference*	
	5-CST > 8.5s	1.19 (1.01, 1.40)	0.034	1.17 (1.01, 1.37)	0.043

Cox proportional hazards model and Fine & Gray models adjusted by age, body mass index, waist circumference, systolic blood pressure, diastolic blood pressure, smoking status, drinking status, comobidities (hypertension, dyslipidemia, diabetes, chronic lung disease, kidney disease), medications (hypertension medication, diabetes medication), laboratory measurements (white blood cell, triglycerides, high-density lipoprotein cholesterol, glucose, cystatin C, hemoglobin A1C, estimated glomerular filtration rate). CI, confidence interval; CVD, cardiovascular disease; 5-CST, 5-time chair stand test; Fine-Gray model, Fine-Gray subdistribution hazard model; HR, hazard ratio; SHR, subdistribution hazard ratio.

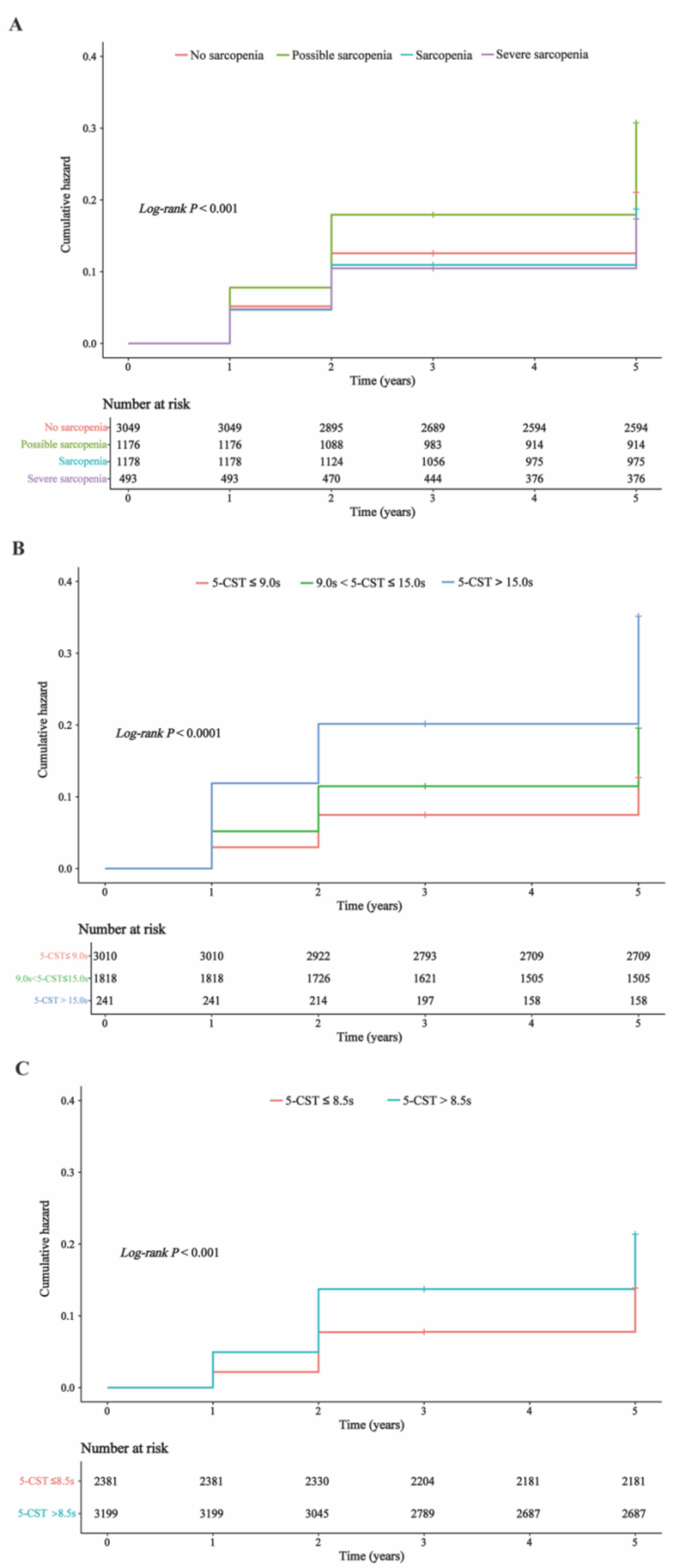

Figure S1. Kaplan-Meier curves for cumulative incidence of composite cardiovascular disease. (A) Stratified by sarcopenia status; (B) Stratified by newly stratified 5-CST categories in males; (C) Stratified by newly stratified 5-CST categories in females. 5-CST, 5-time chair stand test.
